# Synthesis and expression of CDw75 antigen in human colorectal cancer

**DOI:** 10.1186/1471-2407-9-431

**Published:** 2009-12-10

**Authors:** Clotilde Costa-Nogueira, Susana Villar-Portela, Elisa Cuevas, Emilio Gil-Martín, Almudena Fernández-Briera

**Affiliations:** 1 Department of Biochemistry, Genetics and Immunology, Faculty of Biology, University of Vigo, Vigo, Spain; 2 Pathology Service, University Hospital Complex, Ourense, Spain

## Abstract

**Background:**

Increased ST6Gal I activity has been associated with the α(2,6)sialylation enhancement of membrane glycoconjugates observed in metastatic colorectal carcinomas (CRC). Siaα(2,6)Galβ(1,4)GlcNAc sequence, known as CDw75, is a sialylated carbohydrate determinant generated by the ST6Gal I. This epitope has been reported to be associated with the progression of gastric and colorectal tumours, hence there are only a few conclusive studies to date.

**Methods:**

By radioisotopic techniques we evaluated the ST6Gal I activity in healthy, transitional and tumour tissues from 43 patients with CRC. By immunohistochemistry we assessed the CDw75 expression in 25 colorectal adenomas, 43 tumours, 13 transitional and 28 healthy tissues of CRC patients.

**Results:**

ST6Gal I activity was likewise found to be statistically higher in tumour tissue respect to healthy tissue from CRC patients. CDw75 expression was positive in 20% of colorectal adenomas. Furthermore, 70% of tumour specimens and 8.3% of transitional specimens were positive for CDw75 expression, whereas none of the healthy ones showed the presence of the epitope.

**Conclusion:**

The major contribution of this study is the inclusion of data from transitional tissue and the analysis of CDw75 antigen expression in CRC and in colorectal adenomas, little known so far. ST6Gal I activity and CDw75 antigen expression were increased in CRC. Although their comparison did not reach the statistical significance, a great extent of patients showed both, an enhanced tumour ST6Gal I activity and an increased CDw75 expression in the tumour tissue. So, these two variables may play a role in malignant transformation. The expression of CDw75 in colorectal adenomas suggests that this antigen may be a tumour marker in CRC.

## Background

The main role of oligosaccharide structures in recognition phenomena is quite well known [1]. Thus, the oligosaccharide antennae with terminal sialic acids, have been related with the adhesive and invasive properties of cancer cells [2]. Sialic acid is commonly found in glycoconjugates as α(2,3)- or α(2,6)-residues linked to galactose (Gal), as α(2,6)-residues linked to N-acetylgalactosamine (GalNAc) or as α(2,8)-residues linked to other sialic acid unit. Biosynthesis of these various linkages is catalyzed by different members of the sialyltransferase family and it depends on the exogenous substrate acceptor. In this sense, the ST6Gal I [β-galactoside α(2,6)-sialyltransferase; EC 2.4.99.1] catalyzes the formation of α(2,6) linkages; however, it specifically targets terminal Galβ(1,4)GlcNAc (N-acetyllactosamine) structures from glycoproteins. There is yet another α(2,6) specific sialyltransferase identified, namely ST6Gal II, but this enzyme seems to prefer oligosaccharides to glycoproteins as acceptor substrates [3].

There is evidence that the ST6Gal I enzyme activity is significantly higher than the ST6Gal II one in colorectal cancers (CRC) [4], and that one of their most significant glycosylation changes is the elevation of ST6Gal I activity in tumour tissues, when compared with the surrounding healthy mucosa [5]. Additionally, several clinical studies over the past few years, have shown that the ST6Gal I activity is further increased by metastases [6] and that this increase is associated with poor prognosis of the patients [7]. Moreover, while increasing levels of the enzyme in CRC are well established (it occurs in almost all samples analyzed), some studies have also reported the ST6Gal I mRNA increase, where it exclusively affected a handful of cases [8-10].

*In vitro *cell culture studies have shown that ST6Gal I is up-regulated by oncogenes such as *ras *[11-14] and that increased enzyme expression drives the enhancement of β1-integrins adhesion receptor α(2,6)-sialylation [13]. Furthermore, Seales *et al*. [15] reported that β1-integrins from colon adenocarcinomas showed increased α(2,6)- sialylation in comparison with integrins from pair-matched healthy epithelial tissues, suggesting that this hypersialylation is correlated with tumour progression.

The enzyme ST6Gal I, synthesizes the Siaα(2,6)Galβ(1,4)GlcNAc sequence, known as CDw75 antigen, a surface molecule in B lymphocytes. Despite there being several studies focused on B lymphocyte CDw75 expression, little is known to date about the CDw75 expression in solid tumours. Nevertheless, several studies conducted on gastric cancer, in which the authors found the role of CDw75 as a marker of malignant transformation, may be taken into account. These reports documented an increase of CDw75 expression in primary tumours and metastatic gastric carcinomas [16-18], as well as a worsening of patient's prognosis [17,18]; remarkably, none of the healthy mucosa showed CDw75 expression. In the case of the CRC, we understand that only Elpek *et al*. [19] have developed a clinicopathologic evaluation of CDw75 expression in tumour tissues where healthy mucosa showed moderate and no expression, respectively. However, there are no conclusive results to date for ST6Gal I activity and CDw75 expression from CRC. There are a few studies focused on ST6Gal I activity in CRC and even fewer ones specifically concentrated on healthy, transitional and tumour mucosa from the same patient. Similarly, little is known of the role of CDw75 in CRC as a tumour marker, and its behaviour in premalignant lesions like adenomas hasn't been elucidated yet. Consequently this pioneering work has been undertaken to elucidate the response of CRC α(2,6)-sialylation as malignancy progresses, by evaluating both, the ST6Gal I enzyme activity and the CDw75 tissue expression in colorectal adenomas and in healthy, transitional and tumour CRC specimens from the same patients.

## Methods

### Samples

A total of 43 patients (71.19 ± 1.51 years old) with colorectal adenocarcinoma and 25 patients (64.8 ± 2.76) with colorectal adenomas, were randomly selected. They had been diagnosed and treated at the University Hospital Complex (CHOU, Ourense, Spain) between 1997 and 2006. Clinical information about the patients and clinicopathological parameters of the specimens were obtained by the hospital medical charts and were treated in a confidential and anonymous manner. The study has the positive evaluation of the "Comité Ético de Investigación Clínica de Galiza" (Spain). All procedures involving human samples were performed according to the clinical practices of the "Xunta de Galiza" and followed the tenets of the Helsinki Declaration. All patients involved in the study gave their informed consent to participate.

Colorectal adenomas were obtained by surgical polypectomy. In addition, specimens of healthy (distant at least 10 cm away from tumour), transitional (immediately adjacent to tumour but without microscopic features of malignancy) and tumour tissues, from the same patient, were acquired by surgical intervention. All specimens were fixed in formaldehyde (10% v/v) and embedded in paraffin for immunohistochemistry analysis. Aliquots from the same CRC specimens were separated and frozen at the time of surgery, and stored at -80°C until sialyltransferase activity determinations were carried out.

The fraction of total cell membranes from CRC specimens, used as enzyme source, was obtained according to the process described by López *et al*. [20]. The protein concentration of the final membrane fraction was determined by the bicinchoninic acid protein assay (Sigma, St. Louis, MO, USA) [21], using BSA (Sigma, St. Louis, MO, USA) as standard.

### Sialyltransferase assays

ST6Gal I activity was determined using asialotransferrin (AST) as exogenous acceptor. Transferrin (Sigma, St. Louis, MO, USA) was desialylated by mild acid hydrolysis (0.1 N H_2_SO_4_, 1 h, 80°C), according to the TBA method for checking the process [22].

The sialyltransferase assay was carried out according to Vázquez-Martín *et al*. [23]. The reaction mixture, in a total volume of 100 μL, contained: NaF 4 mM, MnCl_2 _5 mM, Triton X-100 0.2% (v/v), MES buffer 40 mM (pH 6), 50 μg enzyme solution, 0.9 mg AST and 200 μM CMP-NeuAc [1 μM CMP-^14^C-NeuAc (specific activity 9.2-12 GBq/mmol, 77.4-80.32 μM, Amersham Biosciences, Uppsala, Sweden) and 199 μM CMP-NeuAc (Sigma, St. Louis, MO, USA)] as donor substrate. The sialylated proteins from the reaction were collected on Whatman glass filters. Radioactivity was measured in a Wallac 1409-12 Scintillator system using Ecoscint H (National Diagnostics, Atlanta, GA, USA) as scintillation cocktail. Endogenous acceptor assays were carried out by incubating standard cocktail reaction without the exogenous acceptor (AST). Enzyme activity was expressed as μU/mg of protein (U, international unit of enzyme activity; quantity of enzyme that catalyzes the conversion of 1 μmol of substrate per minute).

### Immunohistochemical analysis

The immunohistochemical staining was performed with a monoclonal mouse antibody against human CDw75 epitope (Clone LN-1, NeoMarkers, Fremont, CA, USA). Sections (2-3 μm) of selected paraffin embedded tissue blocks from healthy, transitional and tumour specimens of CRC patients and colorectal adenomas, were deparaffinized in xylene, rehydrated in a graded ethanol series, and heated in a microwave oven for 15 min (with citrate buffer 10 M, pH 6.0) to retrieve antigens. Endogenous peroxidase activity was blocked with a blocking solution (Peroxidase block, EnVision™ Detection System, Dako, Carpinteria, CA, USA) for 15 min. To avoid non-specific unions, the tissue slides were exposed for 20 min in BSA. The sections were then incubated with the primary antibody for 30 min (dilution 1/25) in a moist chamber. After a rinse with PSB, the incubation with the secondary antibody bound to peroxidase (goat anti-mouse, labelled polymer, HRP, EnVision™ Detection System, Dako, Carpinteria, CA, USA) was carried out for 30 min in a moist chamber. The immunostaining was visualized with 3,3'-diaminobenzidine reagent. Finally, the sections were counterstained with haematoxylin, dehydrated through graded ethanol series and xylene, and mounted. Negative controls were performed by substituting the primary antibody with PBS.

The evaluation of the immunohistochemical staining, detected as a brown colour precipitate, was carried out independently by two expert pathologists (CHOU), who reached a final result by consensus. The staining pattern was classified semi-quantitatively as follows: 0, tissues without staining; 1, less than 10%; 2, 10-50%; and 3, more than 50% of the tissue stained.

### Statistical Analysis

The statistical analysis was carried out using the SPSS program (14.0 version). Univariate analysis for categorical data was conducted by means of the χ^2 ^test or the Fisher's exact probability test. For continuous data, we employed the Wilcoxon's test, the Mann-Whitney U test and the Kruskall-Wallis' test. Moreover, we performed *a posteriori *test, the Dun test, when marginally significant *p *values obtained suggested the existence of significant differences between groups. Finally, the association between ST6Gal I activity and CDw75 expression from the same specimen was tested using the Spearman's correlation coefficient test. The results were considered significant when *p *< 0.05.

## Results

### α(2,6)Sialyltransferase activity

Specimens of healthy, transitional and tumour tissues from 43 CRC patients were processed and sialyltransferase activity was determined in the total cell membrane fractions. The experimental conditions specifically measured the incorporation of NeuAc into AST, despising the incorporation onto endogenous acceptors. The incorporation of NeuAc into endogenous acceptors was performed by carrying out 8 independent assays without adding exogenous acceptors, and employing specimens from 8 different CRC patients. Levels of activity obtained were 4.37 ± 1.68 and 5.14 ± 1.77 μU/mg protein for healthy and tumour specimens, respectively. There were no statistically significant differences between tumour and healthy tissues (*p *= 0.91, according to Wilcoxon's test, data not shown), therefore these were discarded in later assays.

**Figure 1:**
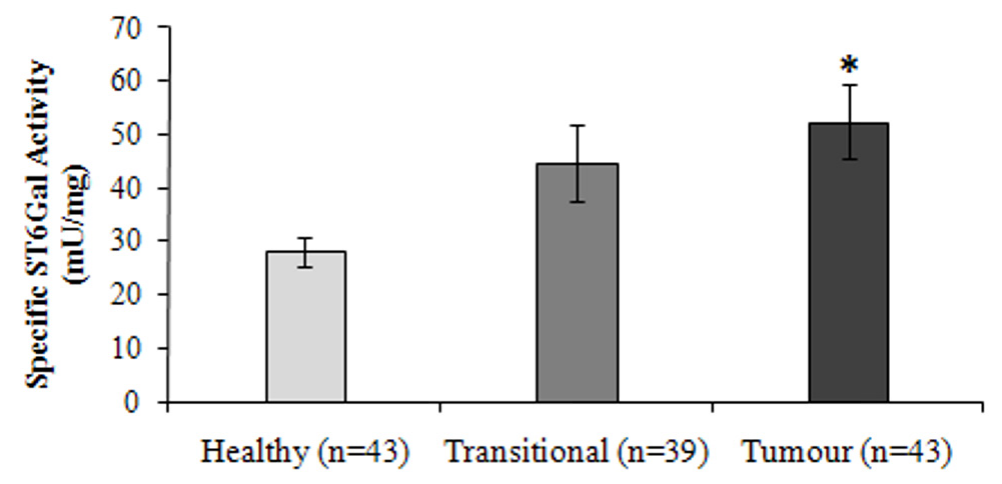
**ST6Gal I activity in healthy, transitional and tumour tissues from the patients with CRC analyzed**. Data are expressed as μU/mg protein and represent the means ± standard error of the means of the *n *independent determinations carried out per duplicate. *p < 0.05 between tumour and its relative healthy tissue according to the Wilcoxon's test.

Figure 1 shows α(2,6)ST6Gal activity measured in healthy, transitional and tumour colorectal tissue specimens from the same patient, using AST as exogenous acceptor and an isotopic mixture CMP-^14^C-NeuAc/CMP-NeuAc as donor substrate. Results showed that ST6Gal I activity in tumour tissue samples was significantly higher than in healthy ones (*p *= 0.003, Wilcoxon's test). The transitional tissue activity was intermediate between healthy control and tumour ones and showed no statistically significant differences.

On the other hand, α(2,6)ST6Gal specific activity in tissues from the 43 CRC patients studied was examined to find correlation with several standard clinicopathologic features. This initial statistical analysis revealed no significant differences with age, tumour location, size, histological grade or growth type (growth type according to Mann-Whitney U test, and the rest according to Kruskall-Wallis' test; data not shown for healthy and transitional tissues, first column of Table 1 for tumour tissue). Nevertheless, as a new strategy to look for statistical differences of tumour ST6Gal I activity, we considered the Tumour/Healthy activities ratio and the Net increase of specific activity in a tumour specimen with respect to the healthy one from the same patient, and performed their corresponding evaluation to find correlations (second and third columns of Table 1, respectively). This new correlation study with the standard clinicopathological features revealed a minor net increase for >77-year-old patients, and a greater ratio tumour/healthy for minor size tumours (Table 1). The remainder of the variables did not reach statistically significant differences (from Kruskall-Wallis' test), but several marginally significant *p *values were obtained suggesting the presence of significant differences between groups. A new statistical study was then conducted by means of the Dun test (Table 2), a post-test which performs multiple comparisons between variable groups. The Dun test showed that differences found regarding the age of the patients were mainly due to the increase of ST6Gal I activity in patients that were < 69 years and > 77 years. So, there is a minor enhancement of activity as patient's age increases. Furthermore, there was an inverse relationship between activity levels and tumour size. Finally, the Dun test revealed a statistically significant difference between moderately and well differentiated tumour histological type, where the former showed higher levels of ST6Gal I activity, of tumour/healthy activity ratio and net increase of activity in tumour specimens (Table 2).

**Table 1:** Relationship between CRC clinicopathologic features and ST6Gal I activity

ST6Gal Activity							
	**Tumor**	***p***	**Tumor/Healthy**	***p***	**Net increase**	***p***	**n**
	**Means ± SEM**		**Means ± SEM**		**Means ± SEM**		

**Age (years)**							
< 69	61.89 ± 13.17	0.887	2.21 ± 0.52	0.981	54.52 ± 12.25	0.042	9
69-77	58.85 ± 15.18		4.25 ± 1.99		62.25 ± 21.74		9
> 77	38.48 ± 6.75		2.60 ± 0.89		20.23 ± 6.04		10
**Tumour location**							
Proximal	41.36 ± 9.28	0.231	2.21 ± 0.87	0.442	28.09 ± 10.11	0.308	7
Distal	72.12 ± 12.58		3.54 ± 1.35		63.46 ± 14.78		13
Rectum	34.56 ± 8.03		2.01 ± 0.69		25.99 ± 9.26		7
**Size (cm)**							
<4	69.89 ± 18.74	0.130	4.96 ± 2.28	0.033	56.86 ± 20.75	0.727	9
4-5	41.45 ± 9.07		1.49 ± 0.320		37.00 ± 13.83		9
> 5	55.81 ± 8.42		3.60 ± 1.17		38.47 ± 6.35		9
**Histologic type**							
Poorly differentiated	8.27	0.208	1.49	0.371	2.72	0.156	1
Moderately differentiated	57.66 ± 7.63		3.27 ± 0.80		49.01 ± 8.99		24
Well differentiated	25.34 ± 0.98		0.80 ± 0.28		2.16		1
**Growth type**							
Polypoid	47.28 ± 10.13	0.915	1.83 ± 0.39	0.414	36.93 ± 11.41	0.716	12
Non polypoid	34.31 ± 11.21		2.75 ± 1.16		23.24 ± 11.56		4
**TNM classification**							
**T**							
T1/T2	76.09 ± 30.13	0.223	3.68 ± 2.25	0.510	74.94 ± 38.60	0.421	3
T3	54.12 ± 8.08		3.12 ± 0.87		43.97 ± 9.65		33
T4	29.00 ± 9.49		1.33 ± 0.58		22.63 ± 19.91		4
**N**							
N0	59.90 ± 10.26	0.139	3.27 ± 1.08	0.443	47.34 ± 11.58	0.491	24
N1	55.83 ± 14.00		3.53 ± 1.50		51.26 ± 16.17		9
N2	27.19 ± 6.90		1.26 ± 0.33		18.07 ± 12.27		7
**M**							
M0	55.76 ± 7.60	0.616	3.16 ± 0.79	0.254	46.42 ± 8.92	-	37
M1	24.93		0.62		-		1
**AJCC classification**							
I	76.09 ± 30.13	0.146	3.68 ± 2.25	0.597	74.94 ± 38.60	0.407	3
IIa	60.05 ± 11.39		3.30 ± 1.27		46.63 ± 12.82		20
IIb	13.17 ± 4.90		0.89 ± 0.59		2.72		2
IIIb	59.70 ± 15.26		3.89 ± 1.66		51.26 ± 16.17		8
IIIc	28.72 ± 7.96		1.42 ± 0.34		18.07 ± 12.27		6
IV	-		0.62		-		1

Relationship between CRC patient's clinicopathologic features and tumour stading and ST6Gal I activity. Tumour/Healthy represents the quotient of the enzyme activity from the two tissues of the same patient. Net increase represents the difference of the enzyme activity from tumour and healthy tissues from the same patient. The ST6Gal I activity and the net increase are expressed as μU/mg protein. Data are represented as means ± SEM (standard error of the means) of the *n *patients analyzed. *p *was obtained from the Mann-Whitney U test (for tumour growth type) and Kruskall-Wallis test (for the rest of variables).

**Table 2:** Comparisons of ST6Gal I activity between clinicopathological features

	Dun Test		
**Clinicopathological features**	**Tumour**	**Tumour/** **Healthy**	**Net increase**

**Age (years)** <69 vs > 77	n.s.	n.s.	7.78
**Histologic type:** Moderately differentiated vs Well differentiated	2.53	6.59	8.24
**T **(T1/T2 vs T3)	4.0	n.s.	1.9
**T **(T1/T2 vs T4)	11	3.6	5.0
**N **(N0 vs N2)	7.0	3.0	5.6
**AJCC **(I vs IIIb)	6.0	2.8	4.9
**AJCC **(I vs IIIc)	5.8	2.6	4.5
**AJCC **(IIa vs IIIb)	7.0	5.2	6.4
**AJCC **(IIa vs IIIc)	6.9	4.8	5.9
**AJCC **(IIa vs IV)	4.4	n.s.	n.s.
**AJCC **(IIb vs IIIb)	5.4	1.8	3.6
**AJCC **(IIb vs IIIc)	5.3	1.6	3.3
**AJCC **(IIb vs IV)	3.5	n.s.	n.s.
**T **(T1/T2 vs T3)	4.0	n.s.	1.9

Comparisons of ST6Gal I activity between clinicopathological features by Dun test. The data represent the value of Dun test, which are indicative of the degree of correlation between the variables. Tumour/Healthy: quotient of the enzyme activity from the two tissues of the same patient. Net increase: difference of the enzyme activity from tumour and healthy tissues from the same patient. n.s.: not significant.

Tumour stage is one of the most important clinicopathologic features and therefore the TNM and AJCC classifications were used, to test the correlation between the tumour stage and both, the tumour/healthy ratio and the net increase of ST6Gal I activity in the tumour tissue of each patient (Table 1). Although the Kruskall-Wallis' test did not reveal statistically significant differences between tumour stage and ST6Gal I activity, a diminished activity was noted in both the TNM and AJCC advanced tumour stages. In this sense, the analysis of the data by means of the Dun test, confirmed that there is a significant decrease in tumour enzyme activity enhancement as the extent of tumour infiltration progresses (Table 2).

### Immunohistochemical expression of CDw75 antigen

**Figure 2:**
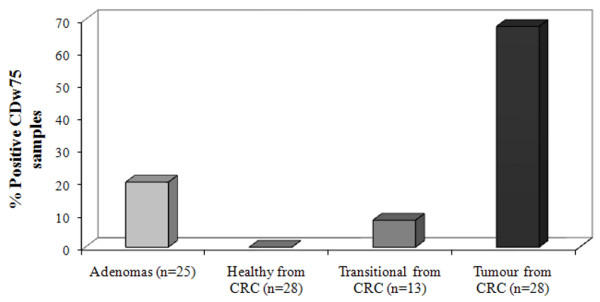
**Percentage of colorectal adenomas and healthy, transitional and tumour CRC specimens which showed positive CDw75 expression in the immunohistochemical study**.

**Figure 3:**
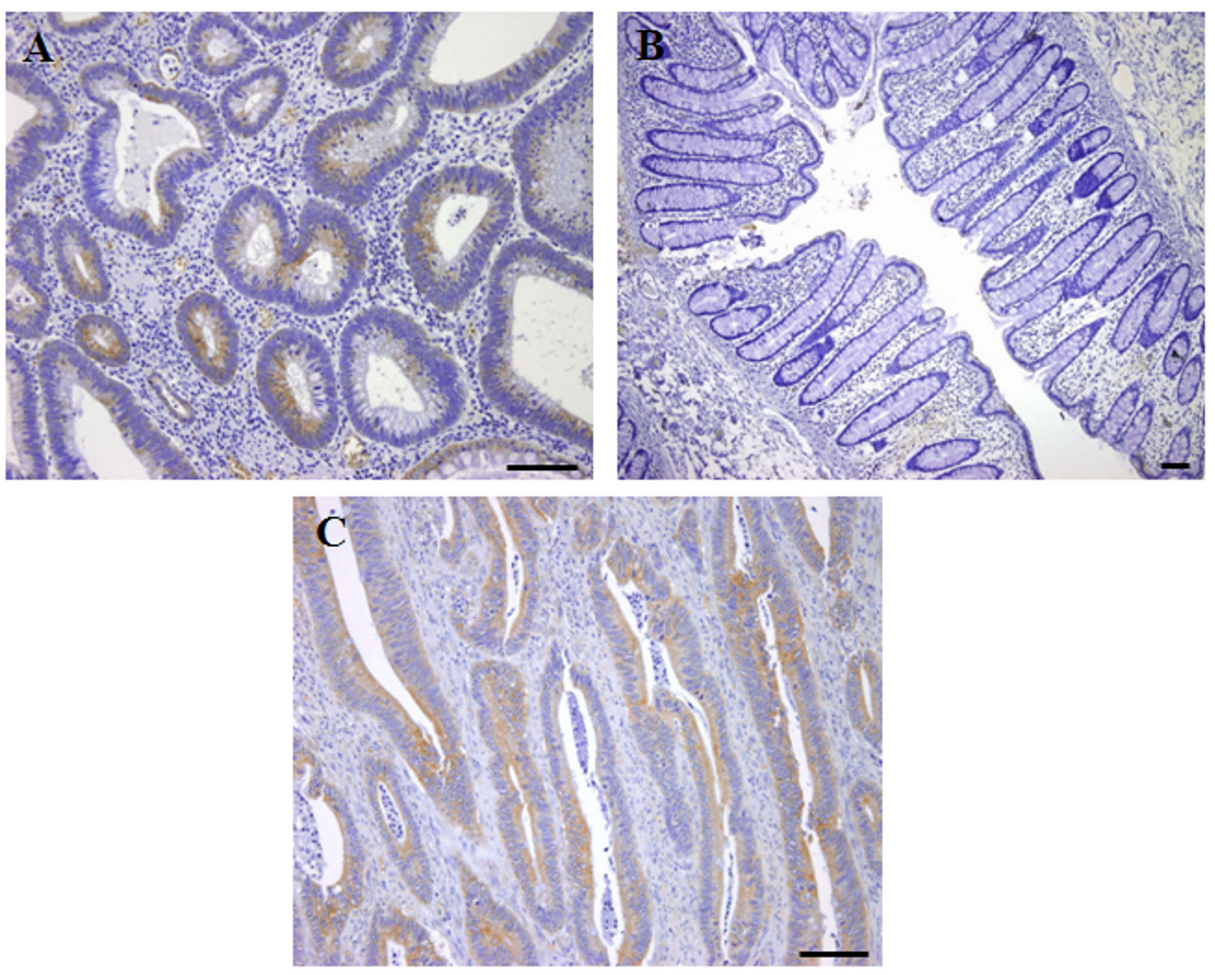
**Immunohistochemical expression of CDw75. **A) Section from colorectal adenomas showing CDw75 expression. B) Section from healthy colon mucosa showing negative CDw75 immunoreactivity. C) Section from CDw75 positive tumour colon mucosa, evidenced by the presence of a brown precipitate. Scale bar = 25 μm.

The CDw75 expression was analyzed in colorectal adenomas and in healthy, transitional and tumour specimens from the same CRC patient. The immunohistochemical analysis showed that the CDw75 antigen was detected in 20% of adenomas (*n *= 25) (Fig. 2). The degree of staining in adenomas was as follows: 12% showed low expression and 8% moderate expression. Likewise, all healthy tissues (*n *= 28) were negative for immunohistochemical CDw75 staining (Fig. 2), while positive staining was detected in 67.9% of the tumour ones (*n *= 28) (Fig. 2), and 8.3% of the transitional mucosa (*n *= 13). Accordingly, when the total of tumour specimens available was considered (*n *= 43), more than 70% of tumour tissues showed positive expression for the CDw75 antigen. The staining intensity was as follows: 9.94% strong, 43.43% moderate and 46.63% low expression. Positive staining of CDw75 was located as a brown precipitate in the cell cytoplasm; staining was diffuse in most cases (Fig. 3). The statistical analysis showed significant differences in CDw75 antigen expression between healthy and tumour tissue (*p *= 0.046, Wilcoxon's test), and between adenomas and tumour specimens (*p *< 0.001, Mann-Whitney U test).

The χ^2 ^test was performed to study the expression of CDw75 antigen in colorectal adenomas and tumours in relation with the clinicopathological features. Results did not demonstrate association between the expression of the CDw75 antigen neither in adenomas nor in CRC and the different clinicopathological parameters considered. Likewise, the degree of expression of the CDw75 antigen as well as the presence or absence of expression, did not seem to correlate, with any of the considered variables (data not shown).

**Figure 4:**
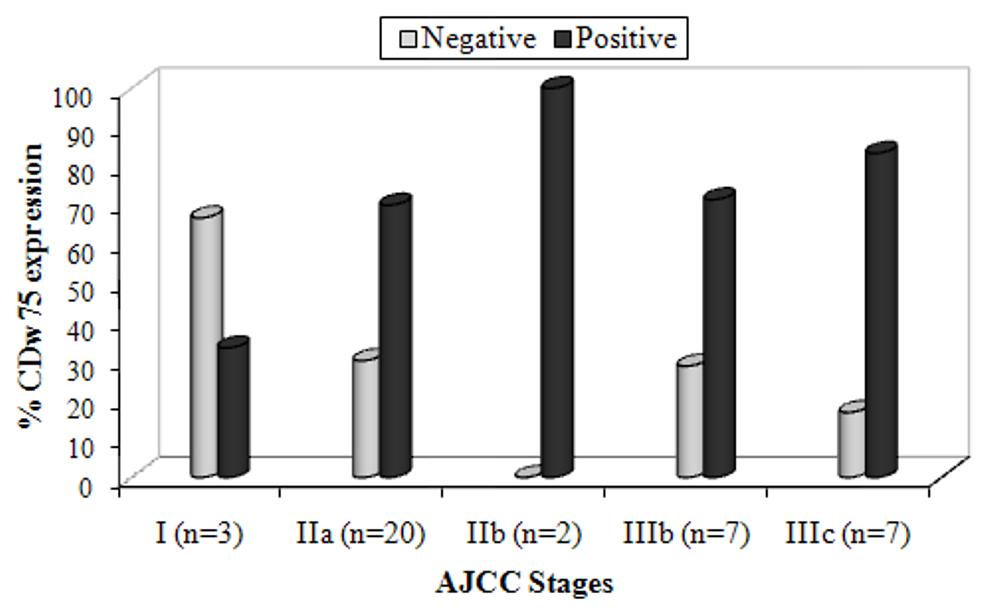
**Percentage of positive and negative CDw75 expression in AJCC stages**. *n *= number of specimens.

TNM and AJCC classifications were used to correlate CDw75 expression (presence or absence and degree of expression) and the tumour stage, and the χ^2 ^test was used to perform the statistical analysis. Although there were no significant differences, highest percentages of CDw75 positive expression in advanced tumour stages were observed (Fig. 4).

### Relationship between CDw75 expression and ST6Gal I activity from the same CRC patient and tissue

The Spearman's correlation coefficient test was carried out in order to correlate both variables, CDw75 expression and ST6Gal I activity from the same patient. The test indicated no correlation (Rho Spearman = 0.106, *p *= 0.505), although in many cases (75%) the two variables showed a similar tissue pattern, such as high-activity and positive expression for the CDw75 in tumour tissue from the same patient.

## Discussion

It can be assumed, that alterations of cell surface sugars change the antigen recognition map of the cells. This fact impacts on interaction patterns among them, as is the case during cell differentiation. Furthermore, glycoconjugates alterations during malignant transformation lead to cell-cell contacts for tissue colonization or the issuance of metastases [24]. Additionally, sialic acid enhancement in the cell surface has been related with a more aggressive behaviour of tumours and an increased metastatic potential [25]. Therefore, depending on the stage of tumour evolution, different disruption levels of glycosyltransferase activities will be responsible for glycoconjugate changes.

The present study detected significant differences between ST6Gal I levels of activity in CRC tumour tissue and the healthy control one. Likewise, it seems that malignant transformation appears in parallel to an upward trend of ST6Gal I activity, since transitional tissue activity values are intermediate between those of healthy and tumour ones. Mostafapour & Goldstein [26] reported that tumour cells with high metastatic potential tend to file a high ST6Gal I activity. Previous studies from our group [23] detected differences between enzyme activity of healthy tissue and transitional ones, in a reduced population of patients. If this difference is confirmed in subsequent analyses, it might seem that tissue cells suffer transitional changes of enzyme activity, perhaps as a prior episode for tumour processing. The current study is one of the few which includes data from transitional tissue, and little is known on this subject so far. Some alterations in transitional tissue have been described regarding its healthy counterpart: sialomucins increase or hyperdevelopment and AG elongation, among others, suggesting that secretor activity is stimulated [27,28]. Despite the foregoing, it is unclear whether the neoplastic process leads to changes in the adjacent tumour mucosa or it is the other way around. So, further and larger studies are needed to clarify the role of α(2,6)-sialylation on CRC origin. In this sense, the assessment of the ST6Gal I activity in premalignant lesions such as colorectal polyps may be useful to elucidate the link between ST6Gal I and CRC development. Nevertheless, the analysis of ST6Gal I activity in colorectal adenomas was not possible because the small size of the resected polyps.

The present study observed that ST6Gal I activity showed a minor increase in the oldest patients, therefore indicating that the enhancement of ST6Gal I is one of the signals of worse prognosis in the CRC. Its largest increase is recorded in the youngest patients, who are likely to develop more devastating tumours. There is therefore a need for a larger population study, in order to clarify the ST6Gal I activity decrease with CRC progression as well as to verify whether the emergence of the disease at advanced ages determines the lower activity.

In as far as tumour size is concerned, as the primary focus grows, the ST6Gal I activity decreases, and therefore the enzyme function would seem most related to training, deployment and initial growth of the tumour through sialylation of the membrane oligosaccharides, than to subsequent tumour growth. On the contrary, we have also noticed that the enzyme activity is higher in such moderately differentiated tumours compared with well differentiated ones.

Upon taking tumour stage into account, we observed that ST6Gal I activity decreased proportionally as the malignancy tumour degree increases. We highlight the presence of statistically significant differences in decline of ST6Gal I activity as it progresses through the infiltration degree or lymph node involvement. The higher ST6Gal I activity in the initial stages is consistent with results from a recent study [29] that reports the changes induced by the expression of ST6Gal I in CRC cell lines that were originally deprived of the same. In this sense, the ST6Gal I activity has been related with the establishment of more partnerships between extracellular substrates recognized by integrins, as well as with an accumulation and differential distribution of β-integrins in the membrane. This study once again demonstrates the role of ST6Gal I in intercellular relationships, and between cells and matrix. Moreover, Seales *et al*. [15], described that an increased α(2,6)-sialylation of β1-integrins in CRC tissues, is more likely to alter interactions of tumour cells with their local matrix environment. Forced ST6Gal I expression in SW48 cells led to increased β1-mediated attachment and migration on collagen I and increased coupling of the β1-subunit to the cytoskeleton-associated protein talin. The manner in which altered the sialylation regulates β1-integrin function is not well understood; however, sialylation could directly affect the ligand-binding activity of collagen receptors [13].

Another focus of attention in our study was the CDw75 expression in CRC. Results showed that the CDw75 antigen [siaα(2,6)Galβ(1,4)GlcNAc], sialylated by ST6Gal I, is expressed in a high percentage of tumour CRC specimens. It is noteworthy that in the case of healthy tissue, 100% of specimens did not show expression for CDw75, and the transitional tissue expression was although significant compared to the no expression in healthy specimens. In this sense, this work is groundbreaking because to date, there are no studies that involve transitional tissue to analyze antigen CDw75 expression. Since the transitional tissue from our study (without histological appearance of malignancy) presents a ST6Gal I activity that is greater than its healthy counterpart, it seems logical to think that sialylation is enhanced and, consequently, that the synthesis of CDw75 antigen, and the probability of detection, are higher. The immunohistochemical and kinetic data included in this study indicate that in the transitional tissue ST6Gal I is indeed expressed, resulting in a significant percentage of cases with positive expression for the CDw75 antigen.

On the other hand, with the aim to elucidate if CDw75 is implicated in the development of CRC, we studied this antigen expression in colorectal adenomas, the precursor lesions of CRC. We found expression of CDw75 in 20% of colorectal adenomas analyzed. The presence of α(2,6)-linked sialic acid residues in colorectal adenomas detected by SNA lectin have been reported by Sata *et al*. [30], and this expression was associated with malignant transformation of adenomas. In our study we have not found correlation between CDw75 expression in adenomas and their malignant potential. It must be considered that the specificity of SNA lectin differs from that of the antibody anti-CDw75, since the first one recognizes both NeuAα(2,6)Gal (CDw75 antigen) and NeuAcα(2,6)GalNAc (STn antigen) sequences [31].

To our knowledge, there is only one previous survey in colon adenocarcinoma, which shows that the CDw75 is not expressed in healthy mucosa but it is expressed in 51.8% of tumour tissues [19]. Moreover, in gastric carcinoma the CDw75 showed similar percentages to those of CRC, 48.4% [17] or 51.4% [18], where the healthy one in both cases was negative. Therefore, these authors have proposed the CDw75 antigen as a molecular marker of gastric carcinoma. Our data showing a very high expression in CRC reinforces the potential role of CDw75 antigen as a marker for malignant transformation.

In the current study, the percentage of CRC specimens found to be positive for CDw75 was higher in tumour CRC advanced stages. Therefore, an increased expression of CDw75 was associated with malignancy or lymphatic invasion, despite the fact that statistical significance was not reached. Elpek *et al*. [19] likewise described highest antigen expression in cases with deeper penetration or advanced tumour stages, as well as in those with metastases. On the other hand, results in literature for gastric adenocarcinoma, have also associated the percentage of CDw75 positive specimens with the tumour stage, the existence of metastasis and the involvement of lymph nodes [17,18]. A lower overall patients survival rate associated with positive expression of antigen has also been described [17,18]. An increase in the expression of CDw75 in tumours with a deeper penetration could be a reflection of increased ST6Gal I activity, especially in tumours with moderate and well differentiated morphology [19]. In fact, positive expression of CDw75 in tumour tissues and absence in healthy ones could indicate that ST6Gal I is increased in CRC. On the other hand, with regards to the tumour infiltration, enzyme activity decreases while the expression of antigen increases. It's very complicated to find correlation between ST6Gal I activity and CDw75 expression. In this sense, Dall'Olio *et al*. have well documented the lack of correlation between the ST6Gal I activity and expression on CRC [32], as well as the non-direct correspondence between the ST6Gal I catalytic potential and the degree of colon glycoprotein α(2,6)-sialylation [33]. The results herein reported also failed in the search for a correlation between the catalytic activity of the ST6Gal I enzyme and the expression of the α(2,6)-sialylated CDw75 determinant. In this sense, it is kwon the existence of multiple ST6Gal I transcripts that account for the post-transcriptional modulation of the enzyme in colon cancer [33,34]. So, it's likely that complex post-transcriptional mechanisms of ST6Gal I have effects on CDw75 expression, and may be the reason of that we haven't found correlation between both variables. Finally, another possible explanation for this result is that the antigen could be sialylated by another sialyltransferase, apart from the ST6Gal I.

We might assume that a greater increase in ST6Gal I activity leads to a higher CDw75 expression. In this sense, it must be pointed out that both variables showed a similar pattern in 75% of CRC specimens.

## Conclusion

In conclusion, this work demonstrates that ST6Gal I activity and CDw75 expression are enhanced in CRC. Likewise, many of tumours studied showed a high ST6Gal I activity and positive expression for the CDw75. Our observations suggest that the two variables may play a role in tumour progression. The expression of CDw75 in colorectal adenomas suggests that this antigen may be a tumour marker in colorectal carcinomas, as proposed in the case of gastric carcinomas.

## Competing interests

The authors declare that they have no competing interests.

## Authors' contributions

CCN carried out the immunohistochemical and enzymatic studies, analyzed and interpreted all data, and drafted the manuscript. SVP participated in the immunohistochemical studies, analyzed data, and was involved in revision of the manuscript. EC collected the clinicopathological data and reviewed the slides. EGM and AFB conceived and designed the study and coordinated its implementation, interpreted data and were involved in critical revision of the manuscript. All authors read and approved the final manuscript.

## Pre-publication history

The pre-publication history for this paper can be accessed here:

http://www.biomedcentral.com/1471-2407/9/431/prepub
